# Impact of Tray and Freeze Drying on Physico-Chemical and Functional Properties of Underutilized *Garcinia lanceifolia* (*Rupohi thekera*)

**DOI:** 10.3390/foods14040705

**Published:** 2025-02-19

**Authors:** Aradhana Boruah, Pinku Chandra Nath, Prakash Kumar Nayak, Maharshi Bhaswant, Sangeeta Saikia, Jatin Kalita, Sarvesh Rustagi, Ajita Tiwari, Kandi Sridhar

**Affiliations:** 1 Department of Agricultural Engineering, Assam University, Silchar 788011, India; 2 Research & Development Cell, Manav Rachna International Institute of Research and Studies, Faridabad 121004, India; 3 Department of Food Engineering and Technology, Central Institute of Technology Kokrajhar, Kokrajhar 783370, India; 4 New Industry Creation Hatchery Center, Tohoku University, Sendai 9808579, Japan; 5 Center for Molecular and Nanomedical Sciences, Sathyabama Institute of Science and Technology, Chennai 600119, India; 6 BioNEST, CSIR–North-East Institute of Science and Technology, Jorhat 785006, India; 7 Center for Infectious Diseases, CSIR–North-East Institute of Science and Technology, Jorhat 785006, India; 8 Department of Food Technology, Uttaranchal University, Dehradun 248007, India; 9 Department of Food Technology, Karpagam Academy of Higher Education (Deemed to Be University), Coimbatore 641021, India

**Keywords:** *Garcinia lanceifolia*, tray drying, freeze drying, antioxidant activity, antidiabetic property

## Abstract

*Garcinia lanceifolia* Roxb. (*Rupohi thekera*), an underutilized minor fruit from Assam, holds significant potential as it exhibits substantial traditional medicinal properties. However, its preservation and utilization remain limited, necessitating effective processing techniques. This study aimed to compare the impact of tray drying and freeze drying on the physico-chemical, antioxidant, and functional properties of *G. lanceifolia*. Fresh fruits were processed using both methods, followed by detailed analyses of nutritional composition, phytochemical content, antioxidant activity, and functional properties. Freeze drying resulted in greater retention of moisture (12.42 ± 0.81%), protein (4.44 ± 0.19%), carbohydrate content (8.29 ± 0.31 g/100 g), and reducing sugar (1.95 ± 0.12%), along with prominent color quality, while no significant difference in ash content was found for either drying method employed. Phytochemical extraction using different solvents (water, n-hexane, 80% methanol, 80% ethanol, and 80% acetone) revealed that freeze-dried samples extracted with acetone had the highest total phenolic content (634.00 ± 1.73 mg GAE/100 g), while methanol extraction yielded the highest total flavonoid content (382.33 ± 1.52 mg QE/100 g). Tray drying, on the other hand, exhibited superior DPPH and FRAP when subjected to ethanol extract (80.24 ± 0.42% and 83.83 ± 0.46 mg/100 g, respectively) and metal chelation capacity (23.69 ± 2.09%). Additionally, functional properties, such as glucose adsorption capacity and α-amylase inhibition, were found to vary between drying techniques, with freeze-dried samples showing better glucose adsorption and tray-dried samples demonstrating greater α-amylase inhibition. FTIR analysis highlighted distinct structural attributes of bioactive compounds retained through both methods. The findings underscore the potential of freeze drying for nutrient preservation and tray drying for cost-effective applications, paving the way for the industrial valorization of *G. lanceifolia* as a functional food ingredient.

## 1. Introduction

**Figure 1:**
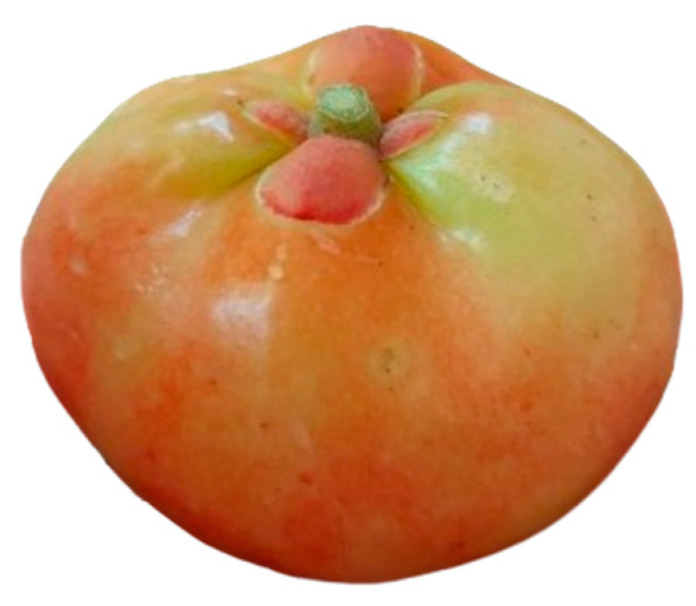
*Garcinia lanceifolia* fruit.

*Garcinia lanceifolia* Roxb., commonly known as “*Rupohi thekera*” in Assam, is an underutilized minor fruit native to the northeastern region of India. *G. lanceifolia* is one of the most important species of *Garcinia* species found in the Brahmaputra Valley of Assam [1] (Figure 1). This fruit, a member of the *Clusiaceae* family, holds immense cultural, nutritional, and therapeutic significance but remains largely underutilized [1]. Traditionally consumed by indigenous communities of Assam for its tangy flavor, *G. lanceifolia* is also recognized for its potential health benefits [2,3]. Fresh and dried slices of the *G. lanceifolia* fruit are used as condiments, for culinary purposes, for the preparation of chutney, pickles, and refreshing drinks, and in traditional medicine for treating a number of ailments, such as digestive problems, dyspepsia, and colic pain [2,3]. Extracts from the fruits have been used to treat digestive ailments, and the bark and leaves have been utilized for their astringent and antiseptic qualities [3]. However, despite its promising nutritional and functional attributes, limited research has been conducted on its compositional analysis and the impact of processing methods on its bioactive profile.

*G. lanceifolia* fruits are highly perishable. Due to the lack of a good post-harvest management system, a bulk quantity of the fruit gets damaged during handling, transportation, and marketing, resulting in a huge amount of annual losses [4]. Drying is a crucial step in post-harvest processing that significantly affects the quality, shelf life, and bioactive compound retention in fruits [5,6]. Many bioactive compounds in fruits, such as ascorbic acid, polyphenols, and flavonoids, are sensitive to high temperatures. Drying at temperatures that are too high can lead to the degradation of these compounds [7]. A temperature of 50 °C is moderate enough to effectively remove moisture but still preserve the integrity of these sensitive compounds.

Fruits are dried using a range of standard drying techniques, including sun drying, solar drying, tray drying, oven drying, cabinet drying, vacuum drying, freeze drying, and many more [8]. However, these drying techniques have a number of limitations. Among the various drying methods, tray drying and freeze drying are widely employed for preserving perishable agricultural products. Tray drying is a frequently employed food preservation process used by both professional food manufacturers and home cooks because of the numerous advantages it offers. Tray drying, which involves conductive heating, is an economical method suitable for large-scale applications. However, the high temperatures involved may degrade thermolabile compounds, such as phenolics and vitamins.

One of the most technologically advanced techniques for dehydration is freeze drying, also known as “lyophilization” [9]. Freeze drying, which utilizes sublimation under low temperatures, is known for its superior ability to preserve the bioactivity of sensitive compounds. This method ensures minimal loss of nutrients [10]. It also helps in the preservation of sample structure and texture, maintaining the natural appearance and integrity of plant-based food samples. Another key benefit is the easy reconstitution of the sample, allowing it to regain its original properties quickly when mixed with water or other liquids [10]. Understanding the differential effects of these drying methods on the nutritional and functional properties of *G. lanceifolia* is essential for determining the optimal approach for its industrial processing and utilization.

Therefore, based on the above-mentioned information, the present study intends to determine the physico-chemical, antioxidant, and functional property changes in “*Rupohi thekera*” when subjected to tray and freeze-drying treatments.

## 2. Materials and Methods

### 2.1. Material Collection

The *G. lanceifolia* fruits were collected from Dohotia (latitude: 22°19′ to 28°16′ north; longitude: 89°42′ to 96°30′ east), Jorhat district (Assam) during the growing season of the fruit (May–July). Unblemished and well-ripened *G. lanceifolia* fruits were washed with clean water, and surface water was wiped using tissue paper. Fresh fruits were sealed in a zipped bag and kept in a −80 °C ultra-low-temperature (ULT) freezer. The cleaned fruits were cut into 0.5 ± 0.25 cm slices using a sharp stainless knife, followed by freeze drying (Free Zone Benchtop, LABCONCO, Kansas City, MO, USA) and tray drying at 50 ± 5 °C (MT-141, FINITE Technologies, Ambala Cantt, Haryana, India) until constant weight of the (*G. lanceifolia*) fruit sliced was achieved. The experiments were performed under a controlled humidity level of 70–77% as per the tray dryer specifications and working conditions. The tray drying process involved conductive heating, where heat was transferred directly to the material through the heated surface of the trays. The dried *G. lanceifolia* fruit slices were ground with a mixer grinder. The powdered samples were then kept in an airtight container at −45 °C without exposure to light for further analysis.

### 2.2. Proximate Analysis

To obtain the initial moisture content, pH, titratable acidity (TA), ascorbic acid, and total soluble solids (TSS) of fresh *G. lanceifolia*, 20 fruit samples were used. The freshly harvested *G. lanceifolia* fruit was subjected to measuring pH using a pH meter (Eutech, Merck, Mumbai, India). The TA of freshly harvested *G. lanceifolia* fruit was determined using a titration method, while the ascorbic acid was determined using a dye method [11]. TSSs were determined using a hand refractometer. For both dried powders, 1 g of the powdered sample was dissolved in distilled water to create a solution (1:10 *w*/*v*). The prepared solution was then subjected to measurements to measure the pH, TA, and ascorbic acid. The moisture content was measured for both fresh and dried samples using a moisture analyzer (MX-50, A&D Company, Limited, Toshima-Ku, Tokyo, Japan). Measurements of the total carbohydrate (Anthrone method), ash content, crude fiber and crude protein content were carried out following the method described in AOAC [12], while the reducing sugar was determined using Nelson–Somogyi’s method [13].

### 2.3. Gross Calorific Value (GCV)

The Bomb Calorimeter (AC350 LECO Cooperation, St. Joseph, MI, USA) was used to measure the GCV of the prepared solid samples, following the standard protocol set for the instrument. Sample (1 g) was used to determine the gross calorific value of the fruit.

### 2.4. Color 

The L*, a*, and b* color values of the *G. lanceifolia* were measured using a color measurement spectrophotometer (Hunter ColorLabUltrascan Vis, Reston, VA, USA). L* indicates the degree of darkness or lightness; a* indicates the degree of redness and greenness; and b* indicates the degree of yellowness and blueness [14]. The hues were calculated as follows using Equation (1).

(1) $$Hue=\tan^{-1}\left(\frac{b^{*}}{a^{*}}\right).$$

### 2.5. Mineral Analysis

A modified method given by Akinyele and Shokunbi [15] based on the method of Crosby [16] was used to prepare the sample for mineral analysis. First, 1 g of dried *G. lanceifolia* sample was weighed into a crucible and subjected to ashing in a muffle furnace at 600 °C for 6 h. The ash residue (1 g) was then dissolved in 10 mL of 1 M nitric acid. Then, it was filtered into a 100 mL volumetric flask using ashless Whatman filter paper and brought up to the mark using deionized water. The mineral estimation was performed using an atomic absorption spectrophotometer against known varied concentrations of solutions of standards of the metals.

### 2.6. Sample Preparation for Phytochemical Analysis

The phytochemicals from the powdered samples were extracted using a solvent extraction method tailored to optimize the recovery of bioactive compounds. The powdered samples were mixed separately with solvents in a 1:10 (*w*/*v*) ratio, including water, n-hexane, 80% methanol, 80% ethanol, and 80% acetone, with each selected solvent based on the polarity needed to target specific hydrophilic or lipophilic bioactive components [17]. The solvent mixtures were transferred into Erlenmeyer flasks and subjected to sonication for 5 min to enhance solvent penetration. Subsequently, the samples were macerated at 37 °C for 5 h using a rotary shaker (the Kuhner LT-X, Adolf Kühner AG, Basel, Switzerland) operating at 120 rpm. The resulting extracts were centrifuged (Sigma 2–16 P, Sigma Laborzentrifugen GmbH, Osterode am Harz, Germany) at 1048× *g* for 20 min to separate the supernatant. The supernatants were then collected and stored at −45 °C for subsequent analyses of phytochemical content, antioxidant activity, and antidiabetic properties.

### 2.7. Total Phenolic Content (TPC)

TPC in the *G. lanceifolia* sample extracts was assessed using the method of the Folin–Ciocalteau assay with little modification [18]. For the analysis, 20 µL of each extract, blank, and standard was placed in a different test tube. Each test tube was then filled with 100 µL of Folin–Ciocalteau reagent (1 N) and 1.58 mL of distilled water. Within 8 min, 300 µL of sodium carbonate was added to each test tube. The tubes were incubated at 37 °C for 40 min. A microplate reader (BioTek, Epoch 2, MEDISPEC (I) Ltd., Kolkata, India) was then used to measure the absorbance at 765 nm. A standard curve was generated using gallic acid standard (Sigma-Aldrich, Mumbai, India), with the concentration ranging from 0.1 to 0.2, 0.3, 0.4, 0.5, 0.6, 0.7, 0.8, 0.9, and 1.0 mg/mL. The results were expressed in mg GAE/100 g.

### 2.8. Total Flavonoid Content (TFC)

The aluminum trichloride method was followed to measure the TFC of the *G. lanceifolia* extracts [19]. Briefly, 2.8 mL of deionized water, 0.1 mL of 1 M potassium acetate, 0.1 mL of 10% aluminum trichloride, 0.5 mL of each sample extract, and 1.5 mL of 95% ethanol were combined. The prepared mixture was incubated at room temperature for 40 min. The absorbance was measured at 415 nm using a Microplate reader against deionized water used as a blank. The standard curve was constructed by measuring the absorbance of quercetin (Sigma-Aldrich, Merck KGaA, Darmstadt, Germany) at concentrations of 0.1, 0.2, 0.3, 0.5, 0.6, 0.7, and 0.8 mg/mL. The quercetin equivalent (mg QE/100 g) of the sample was used to express the results.

### 2.9. DPPH (2, 2-Diphenyl-1-picrylhydrazyl) Assay

The DPPH radical technique was used to evaluate the radical scavenging activity of the sample extract [20]. A sample extract of 100 μL was added to 1.4 mL of a 10^−4^ M DPPH radical methanolic solution for the experiment. After 30 min of incubation, the absorbance at 517 nm was measured using a microplate reader against a blank (100 μL methanol in 1.4 mL of DPPH radical solution). The radical scavenging activity was calculated using Equation (2). The results were expressed in terms of % radical scavenging activity.

(2) $$Radical\;scavenging\;acitivity\;(\%)=\left[\left(\frac{Absorbance\;of\;control-Absorbance\;of\;samplet}{Absorbance\;of\;control}\right)\times 100\right].$$

### 2.10. FRAP Assay

The FRAP activity of each sample was determined using the technique mentioned by Benzie and Strain [21]. In brief, 3.0 mL of FRAP solution was combined with 40 μL of sample extract of each aliquot. After 4 min of incubation at room temperature, the absorbance at 593 nm was measured using a microplate reader in comparison to a blank made with pure water. Then, 2.5 mL of a 10 mM 2,4,6-TPTZ [2,4,6-tri(2-pyridyl)-1,3,5-triazine] solution in 40 mM hydrochloric acid, 2.5 mL of 20 mM ferric chloride, and 25 mL of 0.3 M acetate buffer (pH 3.6) were combined to create a new FRAP solution, which was preheated at 37 °C. The ferrous equivalent Fe (II) μmol/100 g of the sample was used to express the FRAP values.

### 2.11. Metal Chelating Capacity (MCC)

The MCC of the *G. lanceifolia* extracts was determined based on the method reported in the literature [22]. First, 200 μL of each sample extract was mixed with 1.0 mL of 0.3 mM ferrozine and 1.0 mL of 0.13 mM ferrous sulphate for the analysis. A microplate reader was used to record the absorbance at 562 nm after the mixture was kept for 10 min to equilibrate at room temperature. All reaction reagents were present in the control, except the sample extract. Higher activity was observed upon decreased absorbance of the reaction mixture. The chelating activity was calculated using Equation (3) and were expressed in %.

(3) $$Chelation\;activity\;(\%)=\left[\left(\frac{Absorbance\;of\;control-Absorbance\;of\;sample}{Absorbance\;of\;sample}\right)\times 100\right].$$

### 2.12. Functional Properties

#### 2.12.1. Glucose Adsorption Capacity

The *G. lanceifolia* extracts (1%) were added to 25 mL of glucose solution (20 mM). The amount of glucose in the supernatant was measured after the mixture was thoroughly mixed, incubated for 6 h at 37 °C in a shaker water bath, and then centrifuged for 20 min at 1864× *g*. The following Equation (4) was used to determine the concentration of bound glucose [23].

(4) $$Glucose\;adsorption\;capacity\;(\%)=\left[\left(\frac{Absorbance\;of\;control\;blank-Absorbance\;of\;sample}{Absorbance\;of\;control\;blank}\right)\times 100\right].$$

#### 2.12.2. Alpha-Amylase Inhibition

The amylase activity inhibition rate was determined using the reported method with potato starch as the negative control [23]. First, 4 mg of α-amylase and 1% of the sample were mixed in 40 mL of 4% potato starch solution. The prepared mixtures were incubated at 37 °C for 30 min. The final glucose content in the mixture was measured using a glucose assay kit (Coral Clinical Systems, Pantnagar, Uttarakhand, India). The amylase inhibitory effect was calculated according to Equation (5).

(5) $$Alpha-amylase\;inhibition(\%)=\left[\left(\frac{Absorbance\;of\;control\;blank-Absorbance\;of\;sample\;extract}{Absorbance\;of\;sample\;extract}\right)\times 100\right].$$

#### 2.12.3. Estimation of Glucose Uptake by Baker’s Yeast (*Saccharomyces cerevisiae*) Cells

A 10% (*v*/*v*) suspension was made in distilled water after the commercial baker’s yeast was repeatedly centrifuged (1048× *g*, 5 min) in distilled water until the supernatant fluids were clear [24]. Then, 100 µL of each sample extract was added to 1 mL of 25 mM glucose solution, and the mixture was incubated for 10 min at 37 °C. Next, 100 μL of yeast suspension was added to initiate the reaction, which was then vortexed and incubated for an additional 60 min at 37 °C. After 60 min, the tubes were centrifuged for 5 min at 728× *g*, and the glucose content in the supernatant was measured. The percentage of increase in yeast cells’ absorption of glucose was computed.

### 2.13. FTIR

FTIR spectra were recorded using a Spectrum 100 (PerkinElmer^®^, PerkinElmer Life and Analytical Sciences, Shelton, CT, USA) over the wavenumber range of 4000 to 400 cm^−1^ at a resolution of 4 cm^−1^. Duplicate measurements were taken for each sample to ensure accuracy and reproducibility. The samples were prepared by mixing the GLDP with KBr to make the pellet in a ratio of 1:4 (sample: KBr) and analyzed in the transmission mode.

### 2.14. Statistical Analysis

All experiments were carried out at least in triplicates. The values are reported as the mean ± standard deviation. The data were analyzed using Student’s t-test to compare the proximate composition of the processed sample. And, Duncan’s multiple range tests were used for comparing tray- and freeze-dried sample parameters for phytochemical and antidiabetic properties using IBM SPSS Statistics 20 at α ≤ 0.05 significance level.

## 3. Results and Discussion

### 3.1. Chemical and Physical Characteristics of G. lanceifolia Fruit

The proximate compositions of fresh *G. lanceifolia* fruit are listed in Table 1. The proximate composition of fruits serves as a reliable predictor of fruit quality, specifically in terms of nutritional value. Thus, proximate composition analysis is a crucial tool for evaluating fruit quality by enabling the identification of optimal fruit varieties, monitoring the effects of processing and storage, and informing nutritional labeling and marketing claims. The fruit exhibits high moisture content (86.98 ± 0.36%), indicating a short shelf life. The biochemical composition of fresh *G. lanceifolia* fruit was characterized by an acidic pH of 2.39 ± 0.14 and considerable TA of 0.15 ± 0.01%. Fresh *G. lanceifolia* is a rich source of ascorbic acid (18.54 mg/100 g), with TSS content at 5.0 ± 0.01° Brix, indicating moderate sugar content. The nutritional profile is further elucidated by the presence of total ash (1.83 ± 0.23%), crude protein (2.61 ± 0.02%), crude fiber (2.30 ± 0.01%), total carbohydrates (5.43 ± 0.08%), reducing sugar (0.51 ± 0.21%), and a gross calorific value of 1.98 ± 0.03 kcal/g. Colorimetric assessment revealed a distinct yellowish-green hue, quantified by L*, a*, b*, and hue values of 53.02 ± 0.09, 10.22 ± 0.22, 9.26 ± 0.23, and 42.18 ± 0.12, respectively.

The fresh fruits subjected to freeze drying for 48 h and tray drying for 18 h exhibited a significant difference at α ≤ 0.05. The proximate composition and color value of *G. lanceifolia* fruit powder (GLFP) dried using two different drying technologies are listed in Table 1.

Tray-dried GLFP exhibited a moisture content of 09.01 ± 0.32%. The pH value was determined to be 3.05 ± 0.12, with a corresponding TA of 0.17 ± 0.01%. The ascorbic acid content was found to be 15.454 ± 0.01 mg/100 g. The total ash, crude protein, and crude fiber were found to be 1.84 ± 0.23%, 3.53 ± 0.45%, and 5.04 ± 0.82% respectively. The total carbohydrate content was determined to be 6.12 ± 0.04%, with a reducing sugar content of 0.69 ± 0.13%. The gross calorific value was calculated to be 2.99 ± 0.04 kcal/g. Colorimetric analysis revealed L*, a*, and b* values of 49.65 ± 0.04, 6.03 ± 0.51, and 6.83 ± 0.41, respectively, with a corresponding hue angle of 48.34 ± 0.08.

The freeze-dried sample exhibited a moisture content of 12.42 ± 0.81%. The pH value was determined to be 2.82 ± 0.04, with a corresponding titratable acidity of 0.16 ± 0.01%. The ascorbic acid content was found to be 14.11 ± 0.01 mg/100 g. The total ash content was 2.05 ± 0.04%. The freeze-dried GLFP displayed elevated levels of crude protein (4.44 ± 0.19%) and crude fiber (3.37 ± 0.20%) expressed on a dry weight basis. The total carbohydrate content was determined to be 8.29 ± 0.31%, with a reducing sugar content of 1.95 ± 0.12%. The gross calorific value was calculated to be 2.74 ± 0.08 kcal/g. Colorimetric analysis revealed L*, a*, and b* values of 65.34 ± 0.01, 18.61 ± 0.63, and 11.29 ± 0.10, respectively, with a corresponding hue angle of 31.50 ± 0.45.

**Figure 2:**
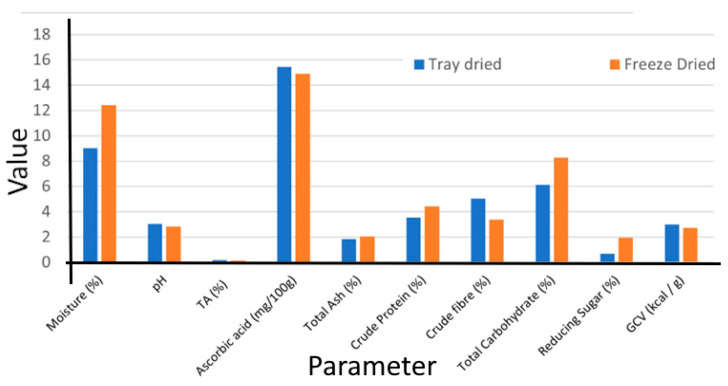
Proximate composition of tray-dried and freeze-dried *G. lanceifolia.* TA, titratable acidity; GCV, gross calorific value.

A comparative graph of the proximate composition of tray-dried and freeze-dried GLFP is presented in Figure 2. The fruit subjected to tray drying methods exhibited a significantly lower moisture content (09.01 ± 0.32%) compared to freeze drying (12.42 ± 0.81%), which may be attributed to the preservation of water-sensitive compounds during the freeze drying process. Furthermore, its acidic character is pronounced, with a pH level significantly lower than neutral. Although differences in drying methodologies were evident, the ash content of the GLFP revealed no statistically significant difference between the different drying methods, indicating that the mineral content remained relatively consistent across drying methods. Tray-dried GLFP exhibits lower crude protein content (3.53 ± 0.45%), which may be because the heat exposure in tray drying results in protein degradation and leads to a perceived reduction in crude protein. Freeze-dried GLFP exhibited a significantly lower crude fiber content (3.37 ± 0.20%), which may be attributed to the prolonged drying time, which causes a breakdown or partial degradation of fibers, leading to a reduction in detectable crude fiber. Furthermore, the total carbohydrate content was found to be higher in the freeze-dried GLFP (8.29 ± 0.31%), likely resulting from the preservation of water-soluble carbohydrates during freeze drying. Similarly, the greatest amount of reducing sugar was observed in the freeze-dried powder. A report on *G. lanceifolia* collected from Mizoram showed considerable variability in physicochemical parameters, and our findings are in agreement with that study [25].

A comparative analysis of the nutrient profiles of GLFP produced using tray and freeze-drying methods demonstrates that both techniques indicate rich compositional quality. Colorimetric analysis revealed that enhanced L* (lightness), a* (redness), and b* (yellowness) values in the freeze-dried GLFP were more prominent in freeze-dried GLFP than tray-dried GLFP. The hue angle (H) values, calculated from the a^*^ and b^*^ coordinates, showed a significant shift in color attributes among the different drying methods, indicating a greater proportion of red tones.

Because no significant difference was found between the different drying methods for the total ash content, GLFP subjected to tray drying at 50 ± 5 °C was used to determine the mineral concentration. The selected minerals determined were Na (sodium), Fe (iron), Mg (magnesium), Cu (copper), Ca (calcium), and Mn (manganese). These concentrations are represented as numerical values of mg/kg of fruit. Fe has the highest concentration at 1.84 ± 0.01 mg/kg of fruit, suggesting that it might be the superior element in the *G. lanceifolia* fruit. However, Cu (0.14 ± 0.02 mg/kg of fruit) has the lowest content, Ca (0.49 ± 0.11 mg/kg of fruit) is present in moderate amounts, and Mn (0.17 ± 0.01 mg/kg of fruit) has a slightly lower concentration. The mineral analysis revealed that the concentrations of the Na and Mg in GLFP were 1.32 ± 0.01 mg/kg of fruit and 0.82 ± 0.02 mg/kg of fruit, respectively.

### 3.2. Effect of Tray Drying and Freeze Drying on Physicochemical Properties and Phytochemical Properties of G. lanceifolia Fruit

**Figure 3:**
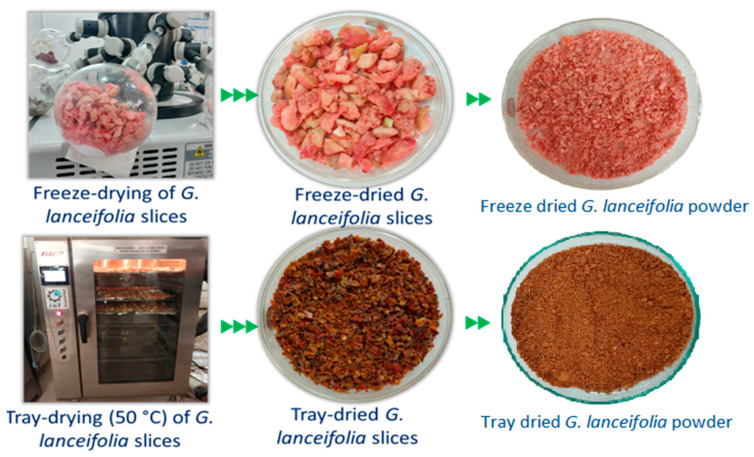
Drying (freeze and tray) of *G. lanceifolia* fruit.

An illustration of the freezing drying and tray drying methods used for drying *G. lanceifolia* fruit, including the dried slices obtained through both drying methods, is shown in Figure 3. Phenolic compounds are the predominant contributors to the overall antioxidant capacity of a matrix, exerting a profound impact on the stability, sensory attributes, and nutritional properties of food products. These bioactive molecules have been shown to significantly mitigate the deterioration of food products by scavenging free radicals responsible for lipid oxidation, thereby preventing the formation of off-flavors, off-odors, and potentially toxic compounds [26]. The antioxidant activity of phenolic compounds has been extensively demonstrated in various studies, highlighting their ability to inhibit radical reactions and protect against oxidative stress [27,28,29]. Furthermore, phenolic compounds have been found to enhance the nutritional value of food products by contributing to their overall polyphenol content, which has been positively correlated with numerous health benefits.

The results of different drying and solvent extraction methods to assess the phytochemical properties of *G. lanceifolia* fruit are presented in Table 2. A similar trend was observed for TPC during the two different drying processes. Notable variation was noticed in the extraction efficiency when different solvent extraction processes were employed. Under freeze drying conditions with acetone extraction, the highest TPC values (634.00 ± 1.73 mg GAE/100 g) were observed, and the lowest TPC value was observed when tray drying at 50 °C employing n-hexane extract (15.33 ± 0.58 mg GAE/100 g). The decrease in TPC could be due to thermal degradation of phenols, as these compounds are very sensitive to heat treatment. However, thermal degradation alone does not explain polyphenol loss at higher temperatures; instead, extraction is significantly influenced by factors like temperature, extraction methods, and the plant matrix [30]. Also, phenolic compounds have a polar character. Non-polar solvents like n-hexane are not very effective at extracting phenolic compounds because phenolics are mostly polar. However, acetone has intermediate polarity. Thus, extraction effectiveness comes from the interplay between the solubilization ability of the solvent and the relative solubility of targeted phenolics from the sample [31]. The highest TPC value was observed with acetone extraction, potentially due to its intermediate polarity, which effectively solubilized and improved the extraction efficiency of the phenolic compound. Research on some phenolic compounds in selected herbs and spices (turmeric, curry leaf, torch ginger, and lemon grass) in 80% acetone, 80% ethanol, 80% methanol, and distilled water extraction systems found that the most efficient extraction of total phenolic compounds occurred with 80% acetone [32].

The highest TFC value was observed with methanolic extraction, followed ethanol and water. A similar trend was observed for both of the drying methods. However, TFC was not detected for n-hexane and 80% acetone extraction for both tray drying and freeze drying. According to research on the chemical structure of flavonoids, their ability to establish hydrogen bonds with solvents has an impact on their solubility [33]. Rutin and isoquercetin are examples of glycosylated flavonoids that exhibit poor solubility in solvents like acetone [33]. Thus, freeze drying powder with methanolic extract gave the highest TFC of value 382.33 ± 1.52 mg QE/100 g. These findings were in agreement with a study on the comparison of the TFC of watermelon rind samples prepared using different drying temperatures (hot-air oven at 40 °C and 60 °C and freeze drying) and extracted using water [34]. They reported that freeze-dried samples had significantly higher TFC than the sample dried using hot-air oven drying at 40 °C and 60 °C. The freeze drying technique provided greater TFC due to its better ability to retain bioactive compounds compared to the hot-air drying method [34].

Likewise, the DPPH and FRAP activity of tray-dried and freeze-dried GLFP was studied. A comparative analysis revealed that powdered samples tray-dried at 50 ± 5 °C show higher antioxidant potency, as measured using DPPH and FRAP assays, compared to freeze-dried samples. The highest DPPH was recorded in tray-dried 80% ethanol extract (80.24 ± 0.42%), and the highest FRAP was recorded in tray-dried 80% ethanol extract (83.87 ± 0.15 mg/100 g). The observed differences in antioxidant capacity suggest that the drying method significantly influences the retention of bioactive compounds. Our results align with previous reports showing enhanced DPPH radical scavenging activity in pepper dried at 60 °C versus 35 °C due to thermal-induced breakdown of free radicals [35]. In addition, moisture content was found to be a critical parameter influencing solvent extraction efficiency of antioxidant compounds, as decreased moisture levels yielded enhanced antioxidant compound recovery [36]. The MCC value was highest in tray-dried water extract. However, MCC was not detected for freeze-dried n-hexane extract or for 80% ethanol or 80% acetone for either of the drying methods. Polar solvents like water, methanol, and ethanol are effective at extracting polar phenolic compounds. These phenolic compounds include functional groups (such as hydroxyl or carboxyl groups) that might bind to metal ions. The variation in results could be due to the presence of other reducing agents, such as ascorbic acid, in the fruits and also genetic, agronomic, cultivar, and environmental factors.

### 3.3. Funtional Properties

**Figure 4:**
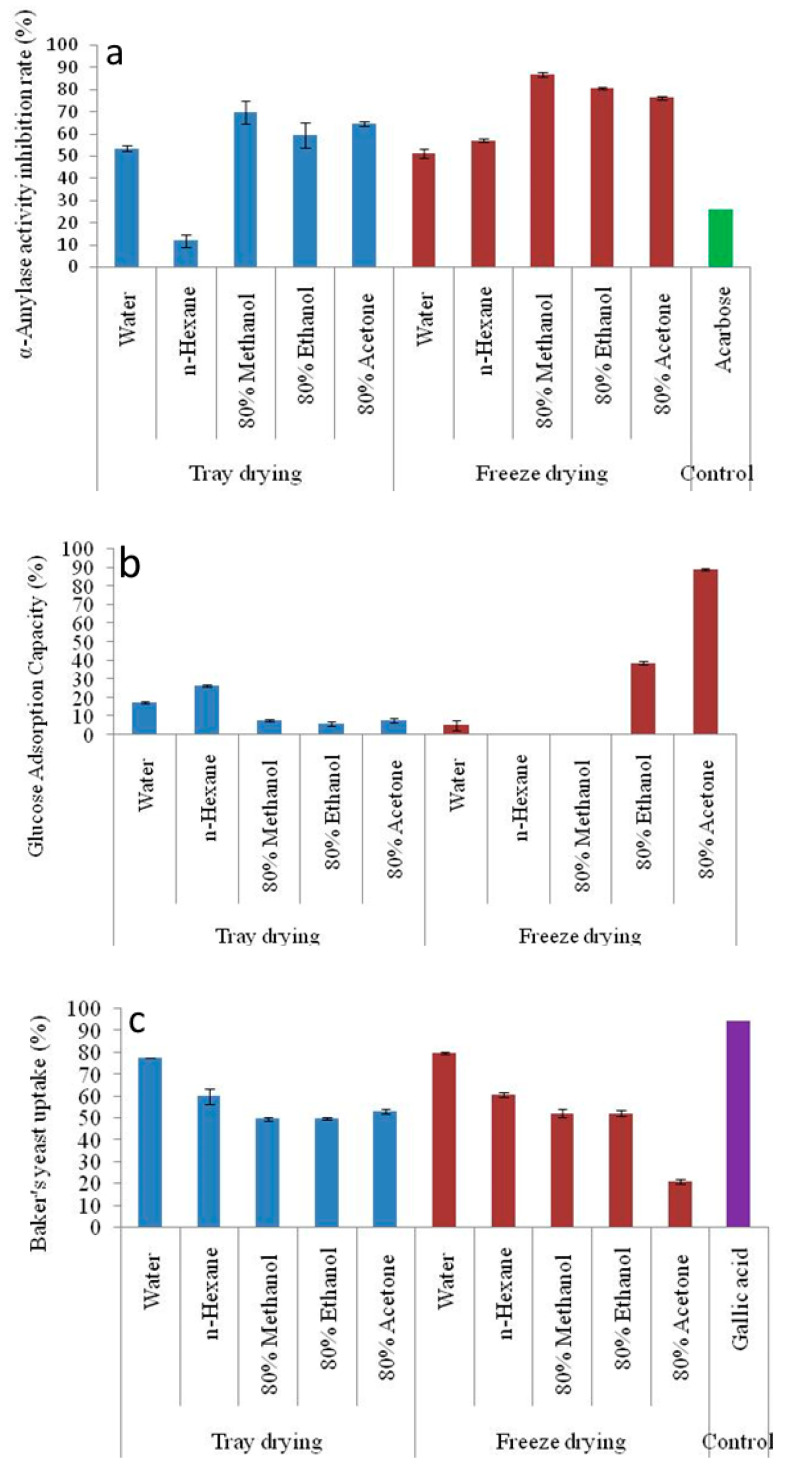
(**a**) α-amylase inhibition, (**b**) glucose adsorption capacity, and (**c**) baker’s yeast uptake of the different solvent extraction and drying methods of GLFP. GLFP, *G. lanceifolia* fruit powder. The error bars represent the standard deviation from the mean of three independent replicates (n = 3).

The α-amylase activity inhibition rate (%) for different solvent extraction and drying techniques is shown against an “Acarbose” control in Figure 4a. An important metric to assess the potential of the extracts to block α-amylase, an enzyme involved in the conversion of starch to sugars, is the inhibition rate of the enzyme. Greater capacity for regulating blood sugar levels is suggested by higher inhibition rates, which is beneficial for controlling the symptoms of diseases like diabetes. The freeze-dried 80% methanol (86.75 ± 0.92%) extract of GLFP exhibited fairly high inhibitory activity against the enzyme compared to the control (26.20 ± 0.01%). However, tray-dried n-hexane (11.69 ± 0.99%) extract showed lower inhibitory activity than Acarbose.

Figure 4b illustrates the glucose adsorption capacity (GAC) of different solvent extracts produced through tray drying and freeze drying. The in vitro ability of freeze-dried and tray-dried GLFP extract to absorb glucose shows significant differences. However, the results showed that freeze-dried GLFP with n-hexane and 80% methanol extract was unable to adsorb glucose. And, among the examined samples, the highest GAC is reported for freeze-dried 80% acetone extract (38.99 ± 0.46%). The higher GAC values for the freeze-dried samples indicate that freeze drying generally retains the structure and bioactivity of the isolated chemicals better than tray drying. However, solvents might affect the efficiency of bioactive compounds when adsorbing glucose, which is essential. Compared to the non-polar solvent (n-hexane), polar solvents like water and ethanol typically have greater capacities for adsorbing glucose. Hydrophilic molecules, which may have greater glucose adsorption capacity, are easier to extract using polar solvents and may have a rich source of hydroxyl- and nitrogen-containing compounds in the form of alcohol/phenol and amide/amines, which have the tendency to inhibit the targeted enzyme due to strong binding on the active site of the enzyme [32,37].

The rate of glucose transport across the cell membrane in the yeast cell system is presented in Figure 4c. Glucose uptake by baker’s yeast cells was included as a preliminary indicator of the antidiabetic potential of *G. lanceifolia*. This assay provides insights into how bioactive compounds retained in the *G. lanceifolia* dried fruit might influence glucose metabolism, which is particularly relevant given the increasing interest in natural products for managing diabetes. The findings of the present study show that the percentage of glucose uptake by yeast cells is significantly affected by different drying methods and different solvent extraction methods. A similar trend of glucose absorption of compounds by (*Saccharomyces cerevisiae*) yeast cell systems is observed for both drying methods. The freeze-dried water extracts exhibit maximum activity (79.55 ± 0.40). The polarity, toxicity, and hydrophobicity of different solvent extracts are some of the elements that determine the complicated interaction between the solvent extract and the absorption of compounds by *Saccharomyces cerevisiae*. And, yeast cells are adapted to the absorption of polar nutrients; the uptake of non-polar substances is frequently restricted.

For the three assays discussed above, the mechanisms underlying the glucose-lowering effect of freeze-dried GLFP extract have been reported. Freeze drying offers several advantages for sensitive samples like fruit powders, particularly in maintaining structural integrity and functional properties [10,38]. The superior bioactivity of freeze-dried GLFP is likely due to the low-temperature processing conditions, which enable maximal retention of bioactive compounds and functional properties. Similar findings regarding *Sargassum fusiforme* processed under four drying techniques (sun drying, hot-air drying, microwave drying, and freeze drying) showed that freeze-dried samples exhibited the highest inhibitory effect against different digestive enzymes [39]. Our findings are in agreement with a report that stated that compounds used against diabetes are mainly found in the aerial parts of the plants and that fruits are effective against diabetes [40].

### 3.4. FTIR Analysis of G. lanceifolia Fruit Powder

**Figure 5:**
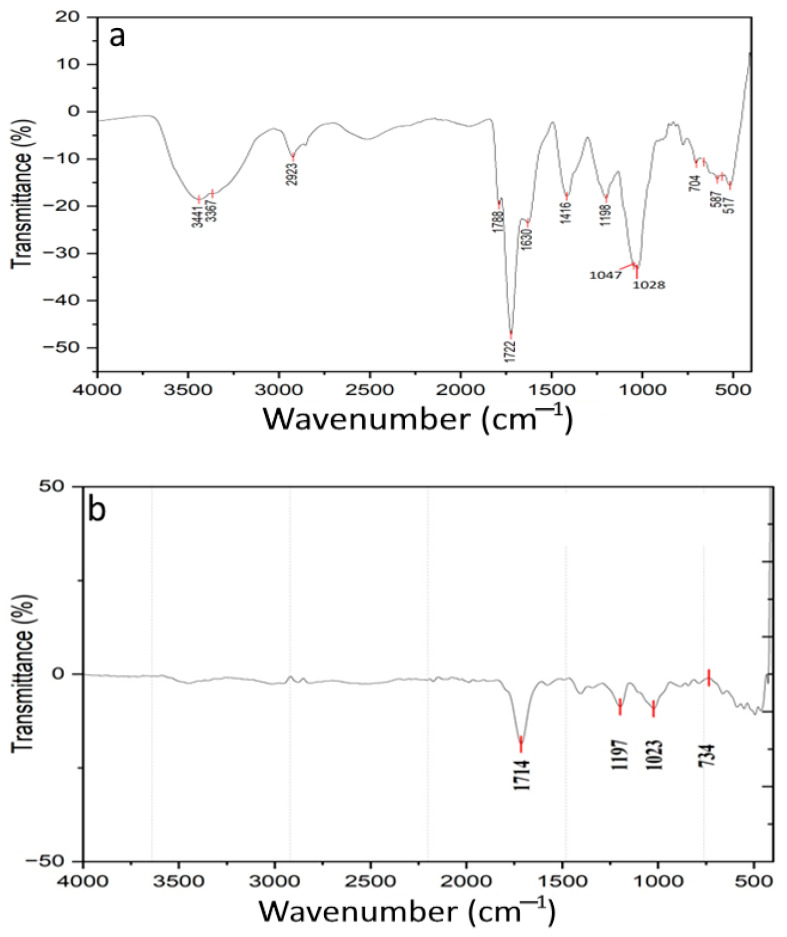
FTIR spectra of GLFP. (**a**) Tray drying and (**b**) freeze drying.

FTIR spectra of tray-dried and freeze-dried GLFP are presented in Figure 5. In Figure 5a, the broad peaks at 3441 cm^−1^ and 3367 cm^−1^ typically correspond to O-H stretching, which is commonly associated with hydroxyl groups (-OH). The peak at 2923 cm^−1^ is often linked to C-H stretching vibration, usually from aliphatic chains like the –CH_2_ group. Peaks at 1788 cm^−1^ and 1722 cm^−1^ likely to correspond to C=O, and the sharpness and position suggest the presence of carbonyl groups, possibly from ester, aldehyde, or carboxylic acid. The peak at 1630 cm^−1^ could be related to C=C, which is often seen in alkenes or aromatic compounds. It may also indicate the presence of amide bands, particularly if the sample contains nitrogenous compounds. The peak at 1416 cm^−1^ is attributed to a C-H or C-O bond, often found in alcohols, ester, or phenolic compounds. And, the peak at the 1198 cm^−1^ and 1097 cm^−1^ region typically corresponds to C-O, which indicates some functional groups related to polysaccharides or esters. The lower wavenumber peaks at 704 cm^−1^, 587 cm^−1^, and 517 cm^−1^ associated with aromatic compounds potentially indicate the presence of a ring structure or may related to halides or metal–oxygen bonds in inorganic compounds.

In Figure 5b, the peak at 1714 cm^−1^ is typically associated with C=O, indicating the presence of a carbonyl group. The peak at 1197 cm^−1^ is often due to a C-O group, typically found in ester, ether, or carboxylic acid. The wavenumber at 1023 cm^−1^ is a region that corresponds to C-O or C-N, which is indicative of ether linkage, alcohol, or amines. C-H bonds or the presence of halides can be interrupted by the peak identified in the region of 734 cm^−1^. This suggests the possible presence of an aromatic ring structure or halogenated compounds. The presence of a polysaccharide related to C-O stretches supports superior functional properties, such as glucose adsorption and α-amylase inhibition. The integration of FTIR data highlights the chemical basis for the functional and bioactive differences between the two drying methods.

## 4. Conclusions

This study compared the impact of two different drying methods, tray and freeze drying, on the physicochemical attributes, antioxidant activity, and functional properties of *G. lanceifolia* (*Rupohi thekera*). Freeze drying retained higher moisture content, crude protein, and total carbohydrates than tray drying. Freeze drying also retained the color (greater lightness, redness, and yellowness) and bioactive compounds extracted with solvents (acetone and methanol). The antioxidant activities, including DPPH and FRAP, and the metal chelating capacity in some of the extracts were also higher for tray-dried samples than freeze-dried GLFP. Freeze drying was more suitable for preserving the nutrient composition and bioactive compounds. In tray-dried samples, greater antioxidant activity was observed. These findings indicate that although freeze drying leads to greater stability of physicochemical properties, tray drying is economical and preserves considerable antioxidant activity, and it could be applicable to broader uses. The extraction of bioactive compounds using both methods for processing *G. lanceifolia* has been shown to have effectively isolated beneficial compounds. However, the drying techniques and the type of solvent used during extraction were critically affected by the retention of bioactive compounds in comparison to their functional properties. Both spectra suggest the presence of organic compounds rich in carbonyl and oxygen-containing groups. The tray-dried GLFP shows complex features, with additional O-H and aliphatic C-H, indicating a more diverse chemical composition. And, the freeze-dried GLFP is simpler, but it still shows clear features of esters or ketones, along with aromatic or halogenated structures.

The present study emphasizes the comparison of tray drying and freeze drying, leaving out other potential drying techniques, such as spray drying or microwave drying. This may affect the bioactive compounds and properties differently. Future work may also be focused on the long-term storage stability of freeze-dried and tray-dried samples to assess the retention of nutritional and functional properties over extended periods for a deeper understanding of how *G. lanceifolia* affects human health, including the specific mechanisms behind the observed antioxidant and antidiabetic activities.

## Figures and Tables

**Table 1:** The proximate composition, calorific value, and color value of *G. lanceifolia* fruit ^1^.

Parameters (Dry Basis)	Fresh *G. lanceifolia* Fruit	Tray-Dried (50 ± 5 °C)	Freeze-Dried
Moisture (%)	86.98 ± 0.36 ^c^	09.01 ± 0.32 ^a^	12.42 ± 0.81 ^b^
pH	2.39 ± 0.14 ^a^	3.05 ± 0.12 ^a^	2.82 ± 0.04 ^a^
Titratable acidity (%)	0.15 ± 0.01 ^a^	0.17 ± 0.01 ^a^	0.16 ± 0.01 ^a^
Ascorbic acid (mg/100 g)	18.54 ± 0.01 ^c^	15.454 ± 0.01 ^b^	14.11 ± 0.01 ^a^
TSS (° Brix)	5.0 ± 0.01	ND	ND
Total ash (%)	1.83 ± 0.23 ^a^	1.84 ± 0.23 ^a^	2.05 ± 0.04 ^a^
Crude protein (%)	2.61 ± 0.02 ^a^	3.53 ± 0.45 ^b^	4.44 ± 0.19 ^c^
Crude fiber (%)	2.30 ± 0.01 ^a^	5.04 ± 0.82 ^c^	3.37 ± 0.20 ^b^
Total carbohydrates (%)	5.43 ± 0.08 ^a^	6.12 ± 0.04 ^b^	8.29 ± 0.31 ^c^
Reducing sugar (%)	0.51 ± 0.21 ^a^	0.69 ± 0.13 ^b^	1.95 ± 0.12 ^c^
Gross calorific value (kcal/g)	1.98 ± 0.03 ^a^	2.99 ± 0.04 ^c^	2.74 ± 0.08 ^b^
Color			
L*	53.02 ± 0.09 ^b^	49.65 ± 0.04 ^a^	65.34 ± 0.01 ^c^
a*	10.22 ± 0.22 ^b^	6.03 ± 0.51 ^a^	18.61 ± 0.63 ^c^
b*	9.26 ± 0.23 ^b^	6.83 ± 0.41 ^a^	11.29 ± 0.10 ^c^
Hue	42.18 ± 0.12 ^b^	48.34 ± 0.98 ^c^	31.50 ± 0.45 ^a^

^1^ Data presented as mean ± standard deviation. ND: not detected. Means with different superscripts in each row significantly differ based on Duncan’s multiple range test (α ≤ 0.05).

**Table 2:** Effect of different solvents on phenolic, antioxidant, and metal chelating activities of *G. lanceifolia* fruit extracts ^1^.

Drying Method	Solvent	TPC (mg GAE/100 g)	TFC (mg QE/100 g)	DPPH (%)	FRAP (mg/100 g)	MCC (%)
Tray drying	Water	358.00 ± 1.00 ^c^	74.33 ± 1.15 ^a^	67.04 ± 1.82 ^d^	77.16 ± 0.06 ^e^	23.69 ± 2.09 ^e^
	n-hexane	15.33 ± 0.58 ^a^	ND	65.01 ± 0.69 ^c^	27.27 ± 0.81 ^a^	1.51 ± 1.09 ^a^
	80% methanol	481.67 ± 2.09 ^e^	341.67 ± 0.57 ^e^	77.01 ± 0.99 ^i^	71.63 ± 0.58 ^c^	10.92 ± 1.00 ^b^
	80% ethanol	495.67 ± 0.58 ^f^	237.33 ± 1.53 ^c^	80.24 ± 0.42 ^j^	83.87 ± 0.15 ^h^	ND
	80% acetone	542.00 ± 1.00 ^h^	ND	75.99 ± 0.89 ^h^	80.30 ± 0.00 ^f^	ND
Freeze drying	Water	464.00 ± 2.08 ^d^	113.33 ± 1.15 ^b^	56.60 ± 0.42 ^b^	74.07 ± 1.78 ^d^	20.22 ± 2.42 ^d^
	n-hexane	29.33 ± 1.15 ^b^	ND	44.04 ± 0.73 ^a^	31.36 ± 0.05 ^b^	ND
	80% methanol	529.00 ± 0.59 ^i^	382.33 ± 1.52 ^f^	69.99 ± 0.64 ^e^	77.90 ± 1.55 ^e^	19.86 ± 1.29 ^c^
	80% ethanol	577.33 ± 2.31 ^d^	233.33 ± 0.58 ^c^	72.95 ± 0.64 ^fg^	81.83 ± 0.46 ^g^	ND
	80% acetone	634.00 ± 1.73 ^j^	ND	73.13 ± 0.28 ^fg^	80.33 ± 0.06 ^f^	ND

^1^ Data presented as mean ± standard deviation. ND, not detected. Means with different superscripts in each column significantly differ based on Duncan’s multiple range test (α ≤ 0.05).

## Data Availability

The original contributions presented in the study are included in the article, further inquiries can be directed to the corresponding authors.

## References

[1] Dutta D., Hazarika P., Hazarika P. (2017). Distribution and Diversity of *Garcinia* L. in Upper Brahmaputra Valley, Assam. Int. J. Curr. Res..

[2] Bora N.S., Bairy P.S., Salam A., Kakoti B.B. (2020). Antidiabetic and antiulcerative potential of *Garcinia lanceifolia* Roxb. bark. Future J. Pharm. Sci..

[3] Angami T., Wangchu L., Singh B., Khonglah L., Thokchom A., Waman A.A., Bohra P. (2021). Chapter-18 *Garcinia lanceifolia* Roxb. (Clusiaceae). Perennial Underutilized Horticultural Species of India.

[4] Mazumdar H., Neog M., Deka M., Ali N., Rajbongshi A., Chakravorty M. (2020). Value Addition of Some Minor Fruits of NE India-A Strategy for doubling farmer’s income. (A review). Int. J. Sci. Res. Publ..

[5] Mohammad A.H., Dey P., Rahman I.J. (2021). Effect of osmotic pretreatment and drying temperature on drying kinetics, antioxidant activity, and overall quality of taikor (*Garcinia pedunculata* Roxb.) slices. Saudi J. Biol. Sci..

[6] Bhattacharjee S., Ramakrishnan E., Deb P.K., Sarma P.P., Choudhury D., Kabilan S., Devi R. (2023). Influence of Drying Condition on Nutritional and Chemical Profile of *Garcinia pedunculata* Roxb. Fruit. Pharmacogn. Mag..

[7] Mphahlele R.R., Fawole O.A., Makunga N.P., Opara U.L. (2016). Effect of drying on the bioactive compounds, antioxidant, antibacterial and antityrosinase activities of pomegranate peel. BMC Complement. Altern. Med..

[8] Hasan M.U., Malik A.U., Ali S., Imtiaz A., Munir A., Amjad W., Anwar R. (2019). Modern drying techniques in fruits and vegetables to overcome postharvest losses: A review. J. Food Process. Preserv..

[9] Indiarto R., Asyifaa A.H., Angiputri Adiningsih F.C., Aulia G.A., Achmad S.R. (2021). Conventional And Advanced Food drying Technology: A Current Review. Int. J. Sci. Technol. Res..

[10] Bhatta S., Stevanovic Janezic T., Ratti C. (2020). Freeze-drying of plant-based foods. Foods.

[11] Dinesh B., Yadav B., Reddy R.D., Sai Padma A., Sukumaran M. (2015). Determination of Ascorbic Acid Content in Some Indian Spices. Int. J. Curr. Microbiol. Appl. Sci..

[12] AOAC (1995). Association of Official Analytical Chemists.

[13] Shao Y., Lin A.H. (2018). Improvement in the quantification of reducing sugars by miniaturizing the Somogyi-Nelson assay using a microtiter plate. Food Chem..

[14] Saikia S., Dutta H., Saikia D., Mahanta C.L. (2012). Quality characterisation and estimation of phytochemicals content and antioxidant capacity of aromatic pigmented and non-pigmented rice varieties. Food Res. Int..

[15] Akinyele I.O., Shokunbi O.S. (2015). Comparative analysis of dry ashing and wet digestion methods for the determination of trace and heavy metals in food samples. Food Chem..

[16] Crosby N.T. (1977). Determination of metals in foods. Analyst.

[17] Alara O.R., Abdurahman N.H., Ukaegbu C.I. (2021). Extraction of phenolic compounds: A review. Curr. Res. Food Sci..

[18] Roy M., Shourove J.H., Singha R., Tonmoy T.A., Chandra Biswas G., Meem F.C., John P.H., Samadder M., Al Faik M.A. (2024). Assessment of antioxidant and antibacterial efficacy of some indigenous vegetables consumed by the Manipuri community in Sylhet, Bangladesh. Heliyon.

[19] Abdullah A.R., Bakhari N.A., Osman H. (2013). Study on the Relationship of the Phenolic, Flavonoid and Tannin Content to the Antioxidant Activity of Garcinia Atroviridis. Univers. J. Appl. Sci..

[20] Brand-Williams W., Cuvelier M.E., Berset C. (1995). Use of a Free Radical Method to Evaluate Antioxidant Activity. LWT Food Sci. Technol..

[21] Benzie I.F.F., Strain J.J. (1996). The Ferric Reducing Ability of Plasma (FRAP) as a Measure of “Antioxidant Power”: The FRAP Assay. Anal. Biochem..

[22] Dinis T.C., Maderia V.M., Almeida L.M. (1994). Action of phenolic derivatives (acetaminophen, salicylate, and 5-aminosalicylate) as inhibitors of membrane lipid peroxidation and as peroxyl radical scavengers. Arch. Biochem. Biophys..

[23] Ou S., Kwok K., Li Y., Fu L. (2001). In vitro study of possible role of dietary fiber in lowering postprandial serum glucose. J. Agric. Food Chem..

[24] Cirillo V.P. (1962). Mechanism of glucose transport across the yeast cell membrane. J. Bacteriol..

[25] Hazarika T.K., Lalnunsangi C. (2018). Exploring genetic diversity of *Garcinia lanceifolia* Roxb. (Clusiaceae), a highly medicinal and endangered fruit of north-east India. Genet. Resour. Crop Evol..

[26] Morandi Vuolo M., Silva Lima V., Maróstica Junior M.R., Segura Campos M.R.E. (2019). Phenolic Compounds. Bioactive Compounds.

[27] Pisoschi A.M., Pop A. (2015). The role of antioxidants in the chemistry of oxidative stress: A review. Eur. J. Med. Chem..

[28] Martins N., Barros L., Ferreira I.C. (2016). In vivo antioxidant activity of phenolic compounds: Facts and gaps. Trends Food Sci. Technol..

[29] Kotha R.R., Tareq F.S., Yildiz E., Luthria D.L. (2022). Oxidative stress and antioxidants—A critical review on in vitro antioxidant assays. Antioxidants.

[30] Antony A., Farid M. (2022). Effect of Temperatures on Polyphenols during Extraction. Appl. Sci..

[31] Gil-Martin E., Forbes-Hernandez T., Romero A., Cianciosi D., Giampieri F., Battino M. (2022). Influence of the extraction method on the recovery of bioactive phenolic compounds from food industry by-products. Food Chem..

[32] Sepahpour S., Selamat J., Abdul Manap M.Y., Khatib A., Abdull Razis A.F. (2018). Comparative Analysis of Chemical Composition, Antioxidant Activity and Quantitative Characterization of Some Phenolic Compounds in Selected Herbs and Spices in Different Solvent Extraction Systems. Molecules.

[33] Rodriguez De Luna S.L., Ramirez-Garza R.E., Serna Saldivar S.O. (2020). Environmentally Friendly Methods for Flavonoid Extraction from Plant Material: Impact of Their Operating Conditions on Yield and Antioxidant Properties. Sci. World J..

[34] Ho L.-H., Ramli N.F., Tan T.-C., Muhamad N., Haron M.N. (2018). Effect of Extraction Solvents and Drying Conditions on Total Phenolic Content and Antioxidant Properties of Watermelon Rind Powder. Sains Malays..

[35] Shotorbani N.Y., Jamei R., Heidari R. (2013). Antioxidant activities of two sweet pepper Capsicum annuum L. varieties phenolic extracts and the effects of thermal treatment. Avicenna J. Phytomed..

[36] Hossain M.B., Barry-Ryan C., Martin-Diana A.B., Brunton N.P. (2010). Effect of drying method on the antioxidant capacity of six Lamiaceae herbs. Food Chem..

[37] Aldughaylibi F.S., Raza M.A., Naeem S., Rafi H., Alam M.W., Souayeh B., Farhan M., Aamir M., Zaidi N., Mir T.A. (2022). Extraction of Bioactive Compounds for Antioxidant, Antimicrobial, and Antidiabetic Applications. Molecules.

[38] Oyinloye T.M., Yoon W.B. (2020). Effect of freeze-drying on quality and grinding process of food produce: A review. Processes.

[39] Zhao T., Dong Q., Zhou H., Yang H. (2022). Drying kinetics, physicochemical properties, antioxidant activity and antidiabetic potential of Sargassum fusiforme processed under four drying techniques. LWT.

[40] Chan C.H., Ngoh G.C., Yusoff R. (2012). A brief review on anti diabetic plants: Global distribution, active ingredients, extraction techniques and acting mechanisms. Pharmacogn. Rev..

